# Chaotic Dynamics of Inner Ear Hair Cells

**DOI:** 10.1038/s41598-018-21538-z

**Published:** 2018-02-20

**Authors:** Justin Faber, Dolores Bozovic

**Affiliations:** 1 Department of Physics & Astronomy, University of California, Los Angeles, California 90095 USA; 2 California NanoSystems Institute, University of California, Los Angeles, California 90095 USA

## Abstract

Experimental records of active bundle motility are used to demonstrate the presence of a low-dimensional chaotic attractor in hair cell dynamics. Dimensionality tests from dynamic systems theory are applied to estimate the number of independent variables sufficient for modelling the hair cell response. Poincaré maps are constructed to observe a quasiperiodic transition from chaos to order with increasing amplitudes of mechanical forcing. The onset of this transition is accompanied by a reduction of Kolmogorov entropy in the system and an increase in transfer entropy between the stimulus and the hair bundle, indicative of signal detection. A simple theoretical model is used to describe the observed chaotic dynamics. The model exhibits an enhancement of sensitivity to weak stimuli when the system is poised in the chaotic regime. We propose that chaos may play a role in the hair cell’s ability to detect low-amplitude sounds.

## Introduction

The auditory system exhibits remarkable sensitivity, for it is capable of detecting sounds that elicit motions in the *Å* regime, below the stochastic noise levels in the inner ear^1^. Fundamental processes that enable this sensitivity have still not been fully explained, and the physics of hearing remains an active area of research^2^.

Mechanical detection is performed by hair cells, which are specialized sensory cells essential for the hearing process. They are named after the organelle that protrudes from their apical surface, and which consists of rod-like stereovilli that are organised in interconnected rows. Incoming sound waves pivot these sterovilli, modulating the open probability of mechanically sensitive ion channels, and thus transforming motion into ionic currents into the cell^3,4^. In addition, hair cells of several species exhibit oscillations of the hair bundle, in the absence of a stimulus^5,6^. These oscillations were shown to violate the fluctuation dissipation theorem and are therefore indicative of an underlying active mechanism^7,8^. The innate motility has been proposed to play a role in amplifying incoming signals, thus aiding in the sensitivity of detection. While their role *in vivo* has not been fully established, spontaneous oscillations constitute an important signature of the active processes operant in a hair cell, and provide an experimental probe for studying the underlying biophysical mechanisms^6^.

The dynamics of an active bundle have been described using the normal form equation for the Hopf bifurcation^9,10^. Several studies have furthermore proposed that a feedback process acts on an internal control parameter of the cell, tuning it toward or away from criticality^11,12^. With the inclusion of dynamic feedback, the theoretical models required three state variables, a dimension that is sufficient to support a chaotic regime, according to the Poincaré-Bendixson theorem. Numerical simulations indeed predicted a small positive Lyapunov exponent, indicative of weak chaos in the innate bundle motion^12^. Another numerical study that explored a 12-dimensional model of hair cell dynamics showed the presence of chaos and proposed that the sensitivity of detection to very low-frequency stimuli would be optimal in a chaotic regime^13^.

The presence of chaos may help to explain the extreme sensitivity of hearing, as it has been shown in nonlinear dynamics theory that chaotic systems can be highly sensitive to weak perturbations^14^. In the present manuscript, we therefore explore experimentally whether innate bundle motility exhibits signatures of chaos^15^. Since establishing the dimensionality of the system is crucial for accurate modelling of this remarkable mechanical detector, we apply a dimensionality test to estimate the number of state variables required to describe the dynamics of an auditory hair cell. Further, we examine the effect of an applied signal on the chaoticity of bundle motion. For this purpose, we construct Poincaré maps of the oscillator, subject to varying amplitudes of external forcing, and test for signatures of torus breakdown. We quantify the degree of chaos by computing the Kolmogorov entropy associated with the spontaneous and driven oscillation of a hair bundle. As a measure of the sensitivity to external perturbation, we compute the transfer entropy from the signal to the oscillatory bundle. Finally, we present a simple theoretical model that reproduces the quasiperiodic and chaotic dynamics that were observed experimentally. We use the theoretical model to demonstrate that a system poised in the chaotic regime shows an enhanced sensitivity to weak stimuli.

## Results

### Dimensionality Test

A useful technique for estimating the dynamical dimension, *d*_*L*_, of a time series relies on the reconstruction of the phase space using delayed coordinates. It has been shown that this delayed-coordinate map from the original *d*_*L*_-dimensional smooth compact manifold M to ${{\mathbb{R}}}^{{d}_{E}}$ is diffeomorphic, provided that *d*_*E*_ > 2*d*_*A*_, where *d*_*A*_ is the box-counting dimension of the original attractor, and *d*_*E*_ is the embedding dimension^16–18^. Frequently, a lower embedding dimension is sufficient to fully unfold the attractor, but it is necessary that *d*_*E*_ ≥ *d*_*L*_^19,20^. In finding the optimal embedding dimension, we set an upper bound on the dynamical dimension of the original dynamics. From the original time series *x*(*t*), we construct the vector,

1 $$\vec{X}=[x(t),x(t+\tau ),x(t+2\tau ),\,\mathrm{...,}\,x(t+({d}_{E}-\mathrm{1)}\tau )],$$

for each point in the time series; *τ* is chosen as the time at which the average mutual information of the series exhibits its first minimum^21^. For each point in the reconstructed phase space, we compute the unit vector,

2 $$\hat{u}({t}_{n})=\frac{\vec{X}({t}_{n+1})-\vec{X}({t}_{n})}{||\vec{X}({t}_{n+1})-\vec{X}({t}_{n})||},$$

where *t*_*n*_ is the time of the *n*^*th*^ observation. These unit vectors point in the direction of local flow on the attractor. For deterministic systems, neighbouring unit vectors are nearly parallel if the attractor is densely sampled, and if the time series is not dominated by stochastic processes. The flow therefore becomes smooth when the embedding dimension is high enough to fully unfold the attractor. The smoothness of this reconstructed phase space can be quantified by finding the average angle that each unit vector (Equation 2) makes with its nearest neighbour^19,22^. Starting with one embedding dimension, we calculate the average angle among all unit vectors, then increase the embedding dimension, and repeat the calculation. The average angle either plateaus or reaches a minimum when using the optimal embedding dimension. The latter case arises when stochastic processes continue to perturb the smoothness in higher dimensions, after the deterministic component has been fully unfolded, thus leading to a gradual increase in the average angle.

**Figure 1:**
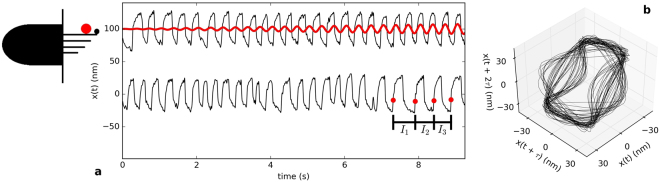
(**a**) Position of a driven hair bundle (top) and spontaneously oscillating hair bundle (bottom). The positive direction corresponds to the direction of channel opening. Red and black traces correspond to the stimulus waveform and the hair bundle response, respectively. The small red dots depict how the time intervals are calculated for the Poincaré maps. The schematic image of a hair cell (left) describes the bundle of stereovilli protruding from the cell body. The large red dot depicts the location of probe attachment to the hair bundle. (**b**) Reconstructed attractor of the hair cell system without stimulus, using delayed coordinates (*τ* = 20 ms).

**Figure 2:**
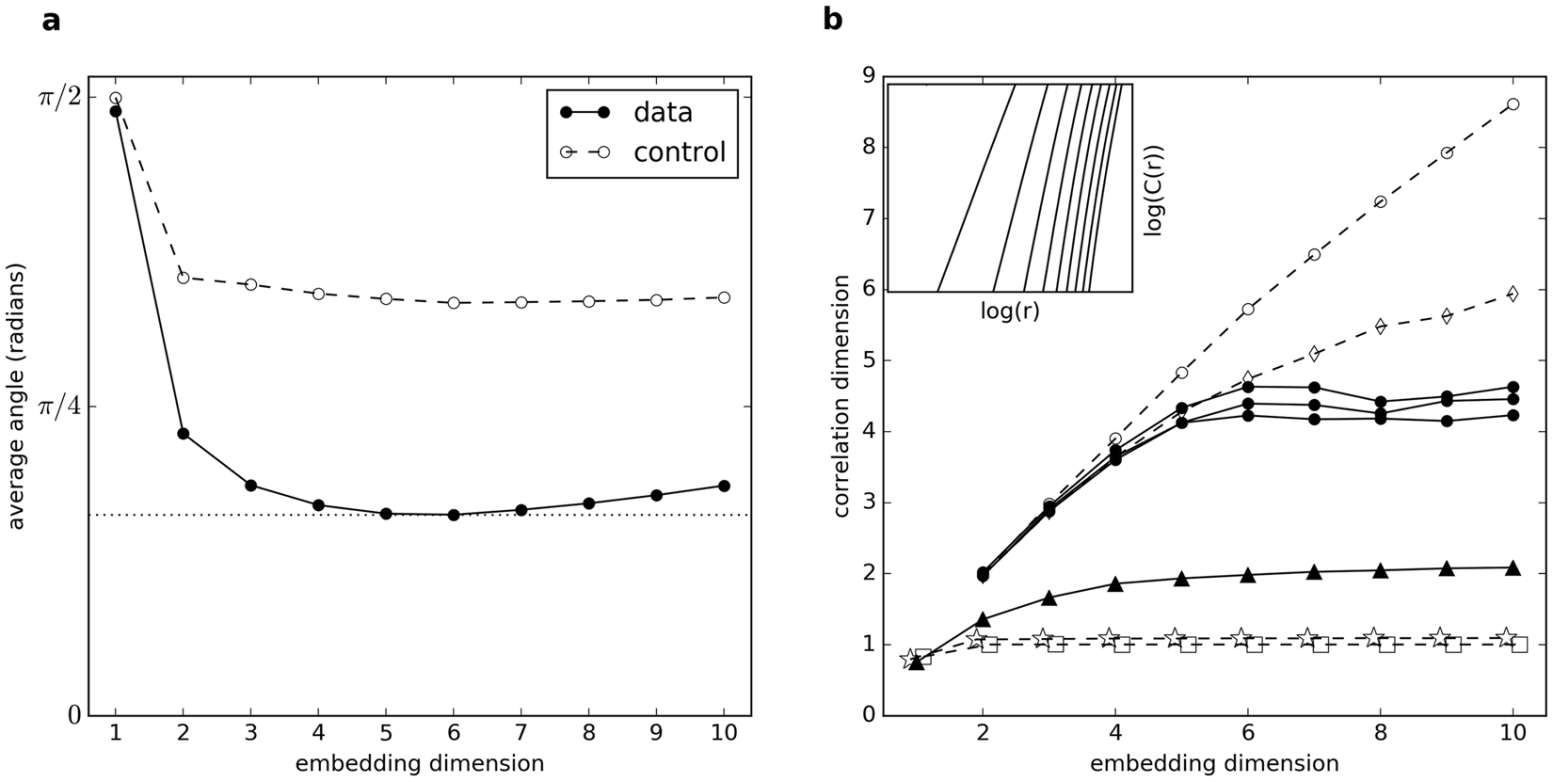
(**a**) Average angle between neighbouring flow vectors as the embedding dimension is varied. The angles are calculated for a 1 minute recording of a typical spontaneously oscillating hair bundle, obtained at 1 kHz sample rate, resulting in 6 × 10^4^ data points. Data were filtered with a low-pass filter to remove high-frequency, stochastic processes. The cutoff frequency was set to 100 Hz, sufficiently above the dominant frequency of the hair bundle (~20 Hz). To maintain a densely-filled phase space, data were not sub-sampled upon filtering. (**b**) Slopes of the extracted linear region vs. embedding dimension for estimation of the correlation dimension. All data sets plotted are of length *N* = 1.8 × 10^5^, unless otherwise specified. “•” and “○” represent raw hair bundle data without stimulus and corresponding phase-shuffled surrogate data, respectively. “▲” represents raw hair bundle data during the torus breakdown, at stimulus amplitude of ~10 pN (*N* = 2.9 × 10^4^). “☆” and “□” represent results obtained from a numerical simulation of a limit cycle with and without 10% additive noise, respectively. “⬦” represents a numerical simulation of telegraphic noise. The inset displays the linear regime, in a log-log plot, of the correlation sum vs. hypersphere radius, averaged over 100 reference vectors for embedding dimensions 2 (left) to 10 (right), computed for the raw hair bundle oscillation data.

For a spontaneously oscillating hair bundle (Fig. 1), the phase space fully unfolds between three and six dimensions (Fig. 2a). As a control, we perform the same analysis on a surrogate data set, generated from the original data as follows. We multiply each Fourier component by a random phase, creating a stochastic signal with the power spectrum and the autocorrelation function identical to those of the original data set. The surrogate data set does not yield a minimum similar to the original data, and the flow along phase space trajectories is much less smooth. We obtained consistent results using an alternate method, referred to in literature as the false nearest neighbour test^23^ (see Supplementary Fig. S3). Further, we performed a direct test for determinism^22^ and found a statistically significant difference between the hair bundle oscillation recordings and their surrogate data sets (see Supplementary Fig. S2). These results indicate that, although stochastic processes are present in our system, there is an underlying low-dimensional attractor. Further, up to six differential equations should be sufficient to describe the dynamic behavior of an active hair cell bundle.

### Correlation Dimension

The fractal dimension of an attractor reflects the space filling capacity of its trajectories. The correlation dimension provides a similar measure and is frequently used to estimate the fractal dimension of a system that is contaminated by noise^24,25^. The correlation dimension can never exceed the number of degrees of freedom of the dynamical system, and hence yields a lower bound. To measure the correlation dimension, the phase space is reconstructed using the delayed-coordinate technique (see section on dimensionality test). Hyperspheres are constructed that are centred on each of the phase space points. The correlation sum is defined as

3 $$C(r)\equiv \mathop{\mathrm{lim}}\limits_{N\to \infty }\frac{1}{{N}^{2}}\sum _{i,j\mathrm{=1}}^{N}{\rm{\Theta }}(r-|{\vec{X}}_{i}-{\vec{X}}_{j}|),$$

where Θ is the Heaviside step function, and ${\vec{X}}_{i}$ is the vector from the origin to the location of the *i*^*th*^ point in reconstructed phase space. The correlation sum is a function of the hypersphere radius, and for small values of r, should obey the power law,

4 $$C(r)\propto {r}^{{\rm{\nu }}},$$

where ν is the correlation dimension. To extract *ν* from the data, we plot $\mathrm{log}(C(r))$ versus $\mathrm{log}(r)$ and find the slope of the linear regime. We repeat this for an increasing number of embedding dimensions, until a plateau occurs in the values of ν. This plateau onset is expected to occur when the embedding dimension exceeds the correlation dimension^26^. This plateau never occurs for a time series dominated by stochastic processes. Integer values of ν imply a non-chaotic attractor while non-integer values of ν are indicative of a chaotic attractor.

In Fig. (2b), we observe a plateau in the correlation dimension, which occurs at a value between 4 and 5. A correlation dimension between 4 and 5 is consistent with our previous results, which indicate that hair bundle dynamics contain between 3 and 6 degrees of freedom. Further, the non-integer correlation dimension suggests a chaotic attractor in the hair cell dynamics. We compare these results to two controls. The first is a surrogate data set generated by shuffling the phases of the Fourier components of the raw data. The second is telegraphic noise generated by solving the Langevin equation in a quartic well potential (see Supplementary Fig. S1). The plateau in correlation dimension does not occur for either of these stochastic data sets. When a sinusoidal stimulus of approximately 10 pN was applied to the bundle, the correlation dimension showed a plateau near 2, consistent with torus breakdown.

### Poincaré Maps

The Poincaré map provides a powerful tool for observing the dynamics of a nonlinear system in a lower dimensional space. For a perfectly periodic signal, the map takes the form of a single point. A quasiperiodic attractor is one whose trajectories densely fill the surface of a torus. The Poincaré map then comprises a ring-like structure which represents a cross section of this torus. The occurrence of trajectories that fall off the surface of this torus indicates a quasiperiodic transition to chaos via torus breakdown^27–31^. Stochastic and high-dimensional processes yield a cloud-like Poincaré map that has no internal structure. The presence of a chaotic attractor could therefore be obscured by the presence of noise.

**Figure 3:**
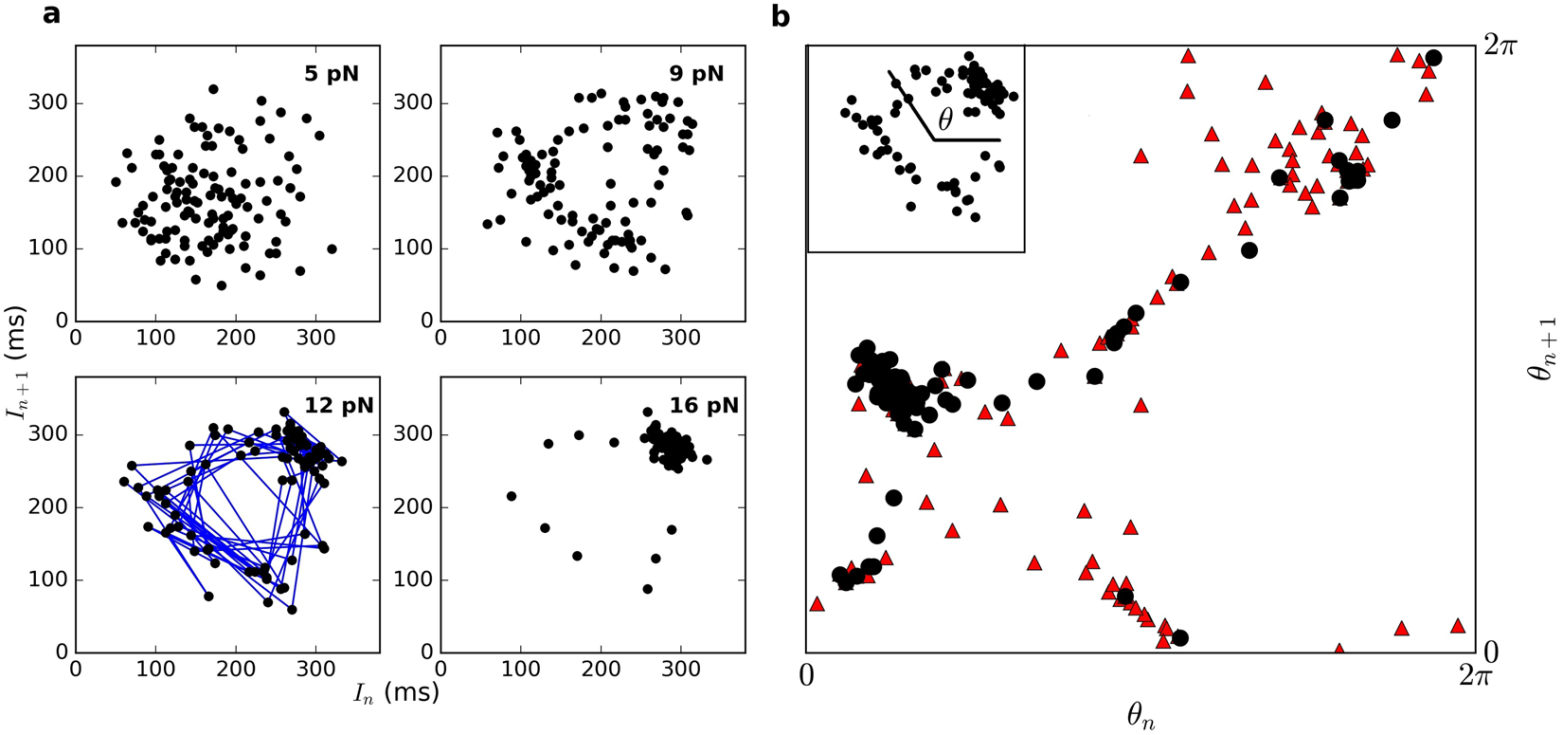
(**a**) Poincaré maps representing oscillations of hair bundles driven by a sinusoidal stimulus below the resonance frequency ($\omega \sim \frac{2}{3}{\omega }_{0}$). Blue lines connect consecutive points in the series. The uncertainty associated with interval measurements is approximately 15 ms (standard deviation). (**b**) Angles formed by vectors from the centre of the ring to each point in the Poincaré map (inset). “” and “•” represent data obtained for stimulus amplitudes of 9 pN and 12 pN, respectively.

Poincaré maps are most commonly constructed by strobing a measured time series at a constant rate^32^. However, this method would not be appropriate for analyzing hair bundle motion, due to the nearly bimodal distribution of bundle positions. As can be seen in Fig. (1a), a typical oscillation approximates a square wave with a varying local period. Hence, to construct Poincaré maps from our recordings, we follow an alternate approach, developed in^33^. We determine the discrete time series, [*I*_*n*_], where subsequent elements are the time intervals between the steepest rising flanks of consecutive bundle oscillations. We then plot the *n*^*th*^ versus the (*n* + 1)^*th*^ point of the series to obtain the Poincaré map. As the series constitutes an observable in phase space, embedding theory can be applied^18^. Poincaré maps constructed for the motion of a hair bundle subject to sinusoidal stimuli at varying amplitudes of forcing are shown in Fig. (3).

At low stimulus amplitudes, and in the absence of stimulus, the Poincaré maps form a cloud-like structure. At higher stimulus amplitudes, a ring structure emerges from the cloud. Consecutive points within the sequence migrate around the edge of the ring, rather than crossing over the centre, indicative of a quasiperiodic behavior. The point density of this ring was found to be significantly distinguishable from surrogate data sets generated by randomizing the order of the elements in [*I*_*n*_]. When the stimulus amplitude is increased above approximately 15pN, the hair bundle follows the stimulus, causing the ring structure to collapse onto a point. This quasiperiodic transition was observed only when the stimulus frequency was below the resonance frequency of the hair bundle.

To test whether the observed quasiperiodic transition corresponds to torus breakdown, a line is drawn from the centre of the ring to each point in the sequence, and the angle formed by these lines and the abscissa is computed. This series of angles yields a circle map, *θ*_*n* + 1_ = *f*(*θ*_*n*_). When chaos arises from a quasiperiodic transition via torus breakdown, points fall off the surface of a 2-torus, since chaotic dynamics can be described by no fewer than three state variables. As a result of the torus breakdown, the map, *f*, becomes noninvertible (multiple *θ*_*n* + 1_ values for a given *θ*_*n*_) and ceases to be a function^34^. As seen in Fig. (3b), the map is non-invertible for a weak stimulus. It approaches an invertible map when a stronger stimulus is applied to the bundle, indicating the disappearance of low-dimensional chaos.

We note that quasiperiodic transitions to chaos exist in multiple forms, reported in different dynamical systems. In our measurements of hair bundle motion, the ring structure in the Poincaré map and the non-invertible circle map together indicate the torus-breakdown route to chaos. The data therefore collectively show that a chaotic attractor exists in the weak stimulus regime, in which the Poincaré map exhibits a cloud. To test the robustness of this analysis, we performed numerical simulations of purely stochastic systems, as well as non-chaotic systems with superposed stochastic processes, and verified that they do not show signatures of torus breakdown (see Supplementary Figs S9 and S10).

We repeated the above experiments at different frequencies of the imposed drive. For stimulus frequencies near the hair bundle’s natural frequency, the Poincaré maps transition directly from a cloud (chaos) to a point (limit cycle), bypassing quasiperiodic dynamics (see Supplementary Fig. S4). For stimulus frequencies above the hair bundle’s resonance frequency, the hair bundle exhibits a flicker between 1:1 and 2:1 mode-locking, over a range of forcing amplitudes (see Supplementary Fig. S6).

### Complexity and Entropy

An additional test for the presence of low-dimensional chaos in a nonlinear system can be obtained from measurements of the permutation entropy and statistical complexity^35–38^. Beginning with a time series, [*x*_1_, …, *x*_*N*_], we take sub-chains of length d, ([*x*_*i*_, …, *x*_*i* + *d* − 1_]). There are *d*! possible permutations of amplitude ordering within the sub-chain (d! different states). A data set of length N produces *N* − (*d* − 1) sub-chains. The probability distribution, P, of these states is used to calculate the normalized Shannon entropy, *H*(*P*),

5 $$S(P)=-\sum _{i\mathrm{=1}}^{i=d!}{p}_{i}\,\mathrm{ln}({p}_{i})$$

6 $$H(P)=\frac{S(P)}{{\rm{\max }}(S)}=\frac{S(P)}{\mathrm{ln}(N)}$$

and the Jensen-Shannon complexity,

7 $${C}_{js}=-2\frac{S(\frac{P+{P}_{e}}{2})-\frac{1}{2}S(P)-\frac{1}{2}S({P}_{e})}{\frac{N+1}{N}\,\mathrm{ln}(N+\mathrm{1)}-2\,\mathrm{ln}\,\mathrm{(2}N)+\,\mathrm{ln}(N)}H(P)$$

where *P*_*e*_ is the probability distribution of maximum entropy (with all states equally probable).

**Figure 4:**
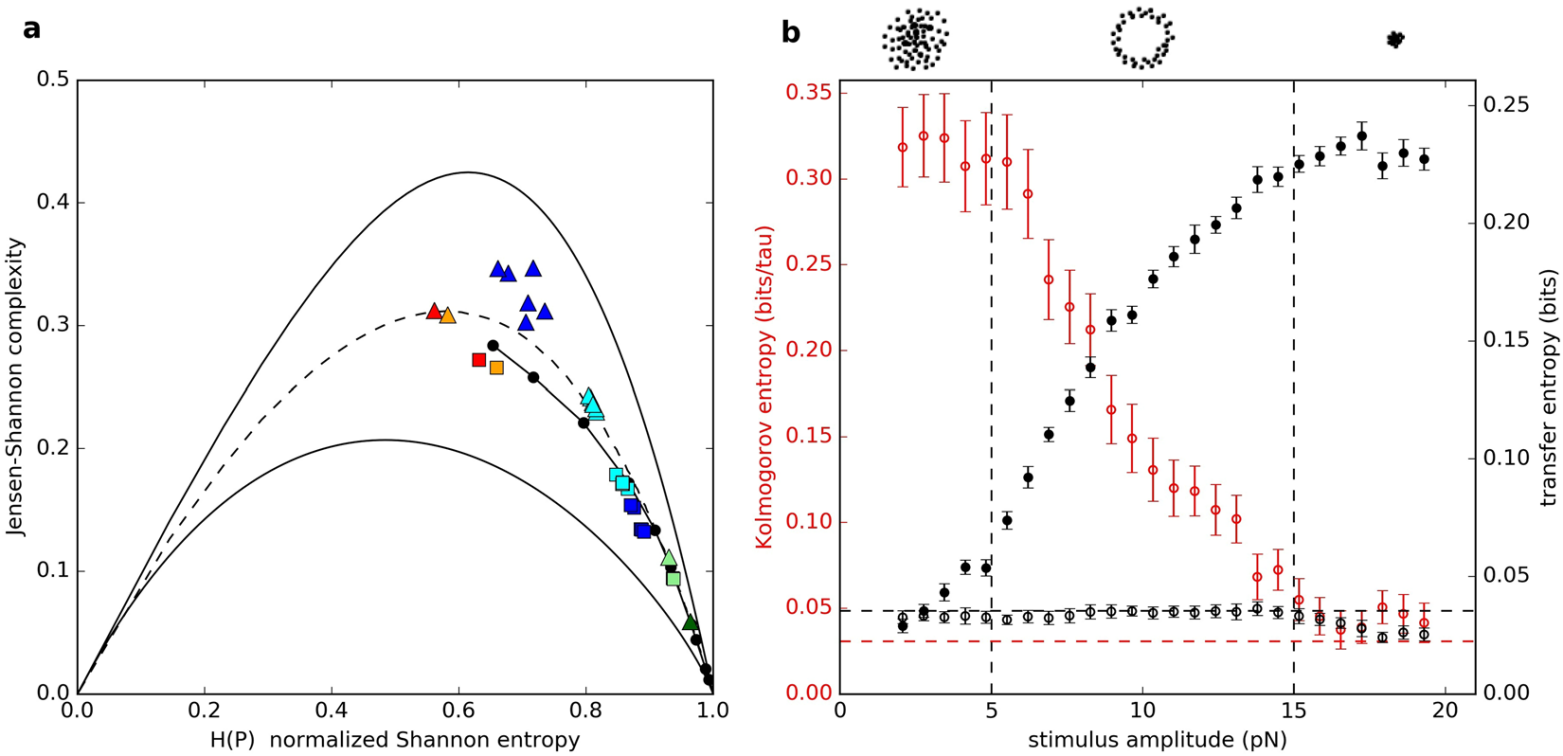
(**a**) Complexity-entropy diagram for the spontaneously oscillating hair bundle in Fig. (2). For all data sets, “■” and “▲” represent fully sampled and sub-sampled data sets, respectively. Blue and teal colours represent raw hair bundle oscillation data and phase-shuffled surrogate data, respectively. Red represents the time series of a numerical simulation of the normal form equation for the supercritical Hopf bifurcations, driven by noise. Orange represents the corresponding phase-shuffled surrogate data set of this Hopf oscillator. Dark green and light green represent telegraphic noise and the corresponding surrogate data, respectively. “•” corresponds to fractional Brownian motion with Hurst exponent ranging from 0.02 to 0.98. The black curves confine all possible probability distributions. The dashed curve is the half-way point between the upper and lower bounds and serves as an approximate boundary between stochastic and chaotic processes. (**b**) The Kolmogorov entropy of an oscillatory hair bundle subject to a sinusoidal drive (“”), and transfer entropy from stimulus to hair bundle (“•”). As a control (“○”), transfer entropy from hair bundle to stimulus is also plotted as a function of the stimulus amplitude. Vertical dashed lines delineate approximate regions that correspond to Poincaré maps displaying cloud, ring, and point structures, as illustrated by the schematic diagrams above the graph. Noise floors are indicated by horizontal dashed lines. The noise floor on the K-entropy is estimated by tracking the motion of a passive hair bundle driven by a 10–25 pN sinusoidal stimulus. The noise floor on the transfer entropy is estimated based on bundle oscillations in the absence of a stimulus. Uncertainties in K-entropy are estimated by taking the standard deviation of the residuals from the linear fit of Shannon entropy with time. Uncertainties in transfer entropy are estimated from a bootstrapping technique that incorporates the uncertainties in position measurements.

All possible probability distributions are confined to a specific region in the complexity-entropy plane. Jointly, the two measurements allow one to determine whether a chaotic attractor or stochastic noise dominates the dynamics of a system. The lower part of the complexity-entropy region (low-complexity) is occupied by probability distributions of stochastic signals, while the high-complexity region is occupied by probability distributions of signals with low-dimensional chaos. We apply this test to our measurements of spontaneously oscillating hair bundles (Fig. 4a). We selected d = 5, which yields 120 (i.e. 5!) possible states. Similar results were obtained for d = 4 and d = 6.

Sub-sampling is a useful technique for extracting a low-dimensional chaotic process from a signal contaminated by noise. A purely stochastic process is hardly affected by sub-sampling; if a chaotic attractor is present in the system, however, it may emerge in the sub-sampled data^39^. Recordings of hair bundle oscillations were sampled at 1 kHz, resulting in a Nyquist frequency of 500 Hz. Sub-sampling every fourth point reduces the Nyquist frequency to 125 Hz, sufficiently above the dominant frequency in the signal (~20 Hz). The sub-sampled data set yields statistical features of low-dimensional chaos (Fig. 4a).

### Kolmogorov Entropy

Kolmogorov entropy quantifies the level of chaos in the trajectory of a dynamical system^40^. Given knowledge of the state of a system at a particular time, obtained with some uncertainty, Kolmogorov entropy (K-entropy) measures how well one can predict its state at a later time. K-entropy therefore provides a measure of how rapidly neighbouring trajectories diverge, and reflects the rate at which the system is producing information. Limit cycles produce no information: for a given observation of the state of the system, all future states can be predicted with the same uncertainty as the original measurement. In contrast, systems exhibiting either low- or high-dimensional chaos constantly produce information. Hence, K-entropy is zero for limit cycles, positive for systems with low-dimensional chaos or noise, and infinite for purely stochastic processes.

To calculate the K-entropy, we divide the reconstructed phase space into hypercubes, and select a starting hypercube to be one that contains many points of the trajectory. We track the trajectories emerging from these points to calculate a time-dependent probability distribution over the hypercubes. The resulting distribution yields the Shannon entropy as a function of time; its time-averaged derivative is defined to be the K-entropy^40–42^. An embedding dimension of five was chosen for this analysis; nearly identical results were obtained with four or six embedding dimensions. The choice of the number of bins per dimension did not affect the results of this analysis. We used two bins per dimension, a natural choice due to the bimodal distribution in position of the spontaneously oscillating hair bundle.

We observe that the Kolmogorov entropy associated with active bundle motility is reduced by the application of a sinusoidal drive (Fig. 4b). The majority of the reduction in K-entropy occurs during the quasiperiodic transition from chaos to order, the regime in which the Poincaré maps produce a ring structure. K-entropy plateaus near zero, for forcing amplitudes above ~15 pN. We note that a noiseless system would plateau exactly at zero; the finite value of the plateau is due to noise inherent in the experimental recording.

Detection by an individual hair cell is quantified by calculating the transfer entropy from the stimulus signal to the receiver response^43^. For systems exhibiting large innate oscillations in the absence of stimulus, we propose that transfer entropy is a more appropriate measure of detection than the traditional linear response function, as it avoids yielding a spurious detection of zero-amplitude signals. Further, in contrast to measures such as mutual information, this measure explicitly identifies the direction of information flow. For a continuous signal, calculation of transfer entropy requires partitioning the range of the signal and assigning a state to each discrete bin. The transfer entropy from process *J* to process *I* is defined as

8 $${T}_{J\to I}=\sum p({i}_{n+1},{i}_{n}^{(k)},{j}_{n}^{(l)})\mathrm{log}\,\frac{p({i}_{n+1}|{i}_{n}^{(k)},{j}_{n}^{(l)})}{p({i}_{n+1}|{i}_{n}^{(k)})},$$

where ${i}_{n}^{(k)}=({i}_{n},\,\mathrm{...,}\,{i}_{n-k+1})$ are the *k* most recent states of process *I*. Therefore, $p({i}_{n+1}|{i}_{n}^{(k)},{j}_{n}^{(l)})$ is the conditional probability of finding process *I* in state *i*_*n* + 1_ at time *n* + 1, given that the previous *k* states of process *I* were ${i}_{n}^{(k)}$ and given that the previous *l* states of process *J* were ${j}_{n}^{(l)}$. The summation is performed over the length of the time series, as well as over all accessible states of processes *I* and *J*. Transfer entropy *T*_*J* → *I*_ is a measure of how much one’s ability to predict the future of process *I*, given its history, is improved by knowledge of the history of *J*.

To analyse experimental recordings of hair bundle oscillations, we discretized the signal into two bins, due to the bimodal distribution in position of the hair bundle; similar results were obtained when using three or four bins. The choice of *k* and *l* had little effect on our results, so we selected *k* = *l* = 4. The transfer entropy from the applied stimulus to the hair bundle rises above zero for signals of ~3 pN. The observed detection threshold is even lower when a stimulus is applied at the resonance frequency of the cell (see Supplementary Fig. S5).

### Theoretical Model

To capture the chaotic dynamics of an oscillatory hair bundle, we apply a variant of a previously proposed model^12^, developed to account for the complex multi-mode hair bundle oscillations. The model was shown to reproduce a number of experimental observations. It captured the sporadic transitions between quiescence and innate oscillation exhibited by hair bundles. Further, it displayed multi-mode phase-locking to the stimulus over a wide range of driving frequencies, in agreement with experimental results. Finally, it reproduced the observed suppression and recovery of oscillation, following strong mechanical forcing. The model consists of three dynamical variables, the minimum dimension capable of supporting a chaotic regime.

The transition between oscillatory and quiescent states is described using the normal form equation of the subcritical Hopf bifurcation (Bautin bifurcation)^15^,

9 $$\frac{dz}{dt}=z(\mu -i{\omega }_{0}+A|z{|}^{2}-B|z{|}^{4})+{f}_{A}\,\cos ({w}_{d}t),$$

where *z*(*t*) describes the dynamic state of the bundle with the real part, *x*(*t*), representing the bundle position. *f*_*A*_, *ω*_*d*_, and *ω*_0_ denote the driving force, driving frequency, and natural frequency, respectively. The fifth-order term in *z* is included to capture the subcritical Hopf bifurcation, which was shown to describe well the complex bundle dynamics^12^. *A* = *B* = 10 and *ω*_0_ = 1, unless otherwise stated.

The control parameter, *μ*, is associated with the probability of the system being in the oscillatory or the quiescent state. We assume the parameter to be a real-valued dynamic variable, with its rate of change described by

10 $$\frac{d\mu }{dt}={k}_{on}-{k}_{off}{\rm{\Theta }}(x-{x}_{0})+\alpha {f}_{A},$$

where *k*_*on*_ and *k*_*off*_ are rate constants. The Heaviside step function, Θ, serves as an approximation of the Boltzmann distribution related to the opening probability of the ion channels^8^. We introduce here the *αf*_*A*_ term to the original model, to capture the entrainment of the bundle by a strong stimulus and to reproduce the torus breakdown transition from chaos as stimulus amplitude is varied. *α* = 0.15 unless otherwise stated.

We demonstrate that numerical simulations based on this model reproduce well the characteristic features of the experimental results. Sinusoidal stimuli of linearly increasing amplitude elicit a quasiperiodic transition from chaos to order via reverse torus breakdown (see Supplementary Fig. S8). This transition is accompanied by a reduction in the Kolmogorov entropy similar to our experimental recordings. We note that higher dimensional models would certainly capture more details of the experimentally measured hair bundle response. However, we aim to find the simplest model that can reproduce the experimental results specific to this study, namely signatures of an underlying chaotic attractor. A simple model has a more tractable parameter space, allowing us to isolate the impact of chaos on the response of the system.

**Figure 5:**
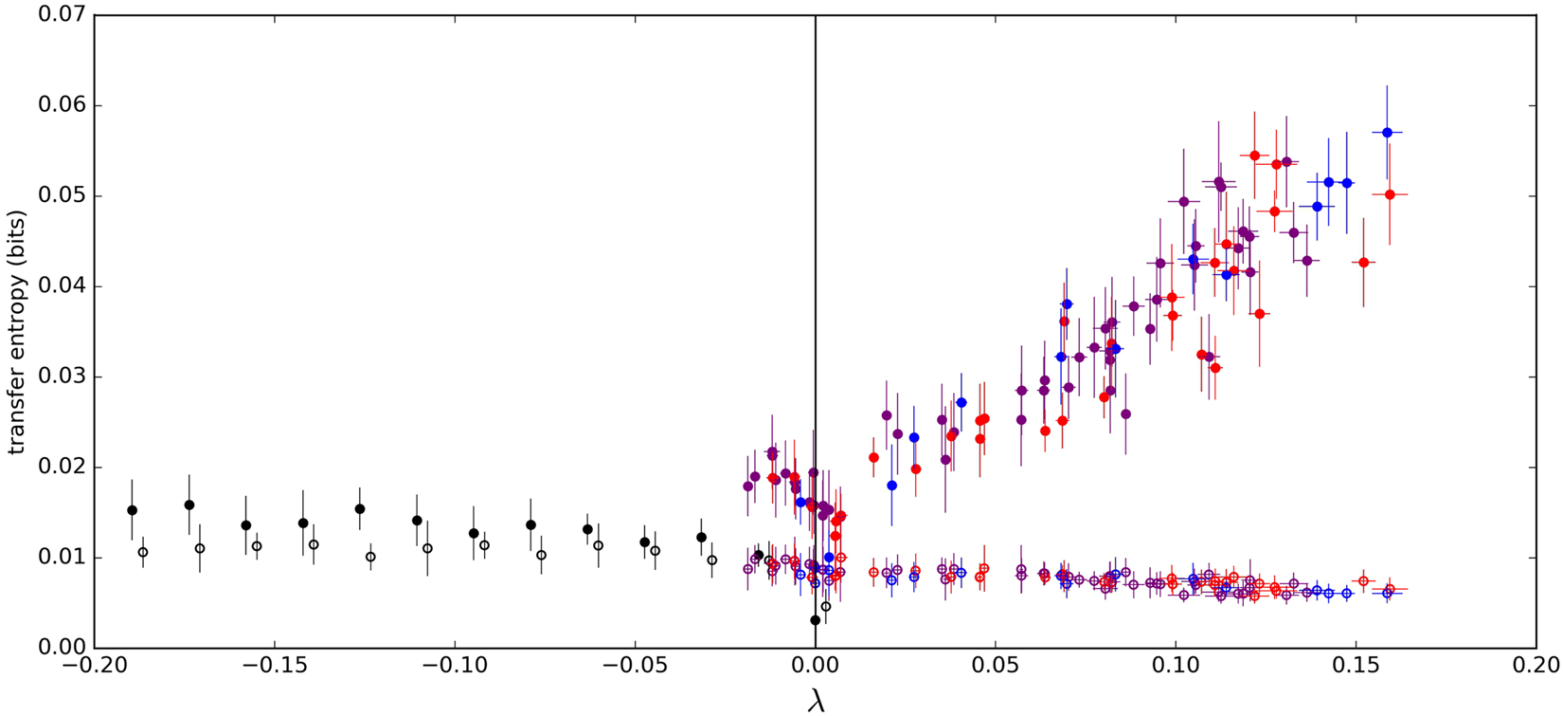
Scatter plot of the largest Lyapunov exponent (*λ*) versus the transfer entropy from a stimulus to hair bundle oscillator, obtained from a numerical simulation. The applied stimulus was a square wave with stochastic variations of the period. The mean of these periods was set to $\frac{1}{2\pi {\omega }_{0}}$ (on resonance) with a standard deviation that is 30% of its mean. The amplitude was set to *f*_*A*_ = 0.3. This numerical value corresponds to forcing amplitude of approximately 1 pN. “”, “”, and “” correspond to variations in parameters *k*_*on*_ (0.3–0.6), *k*_*off*_ (1.0–2.2), and *x*_0_ (0.2–0.7), respectively. “•” represents variations in the cubic parameter, *A* (10^−5^ − 0.15), when the system is poised in the limit cycle regime. In this regime, the Lyapunov exponent of largest magnitude is calculated analytically. As any parameter is varied, all others are held constant (${x}_{0}=\mathrm{0.4,}\,{k}_{on}=\frac{5}{12}$, ${k}_{off}=\frac{7\pi }{12\arccos \mathrm{(0.4)}}$, and *A* = 10.) Error bars in transfer entropy are the standard deviation from repeating the calculation on 10 different stimulus forms, each containing *N* = 10^5^ data points. Open circles of all colours correspond to calculations of transfer entropy in the reverse direct (bundle to stimulus) and serve as controls.

We next explore the effects of chaoticity on the sensitivity to a weak stimulus. In numerical simulations, chaoticity is most easily quantified by the largest Lyapunov exponent of the system^15^. In the absence of a stimulus, Lyapunov exponents are calculated by tracking the rate of divergence of neighbouring trajectories in the 3-dimensional phase space. We vary the parameters of the model, to obtain a broad range of Lyapunov exponents. For each set of parameters, we impose a stimulus signal below, above, and at the natural frequency of the oscillator. For simplicity, we use a square wave stimulus with stochastic variation of the period. We compute the transfer entropy from the imposed stimulus to the hair bundle response, for each of the Lyapunov exponents, and present the results in a scatter plot. For frequencies at or away from the characteristic frequency of the bundle, the model shows enhanced sensitivity to information transfer when poised in the chaotic regime (Fig. 5).

## Discussion

The auditory system has provided an experimental testing ground for theoretical work on nonlinear dynamics^9,11^, nonequilibrium thermodynamics^44^, and condensed matter theory^45^. The fundamental questions on hearing pertain to its sensitivity, frequency selectivity, rapidity of detection, and the role of an active mechanism in shaping the response. Models based on dynamic systems theory have successfully described a number of empirical phenomena. However, the theoretical models have mostly focused on stable dynamics, exploring either the limit cycle regime, or the quiescent regime in the vicinity of a bifurcation.

Our results provide an experimental demonstration that a low-dimensional chaotic attractor arises in the dynamics of active hair bundle motility. Ring-like structures in the Poincaré maps, torus breakdown, positive Kolmogorov entropy, non-integer correlation dimension, and the location of the time series in the complexity-entropy plane are all indicators of low-dimensional chaos, not stochastic processes. Because chaos cannot exist in dynamical systems of dimension lower than three, the two-dimensional models extensively used in the field are insufficient for capturing the dynamics. We estimate that three to six independent variables are needed to correctly characterise the dynamics of the system.

Further, we find that chaos is removed by the application of a signal, with different transitions from chaos to order observed when oscillations are entrained by stimuli below, near, or above the characteristic frequency of the cell. Hair bundles have thus far been viewed as nonlinear mechanical detectors, and the linear response used to characterise their sensitivity. We propose that information theory provides a useful and complementary tool for analyzing the response of a hair cell, which can be viewed as a computational device that serves to extract information about the external acoustic environment. We hence apply an information theoretic approach to quantify the detection of a signal by an individual hair cell. Hair bundles poised in the chaotic regime exhibit measurable increases in transfer entropy even at pN levels of stimulus, indicating that chaotic dynamics of innate motility are consistent with high sensitivity of detection.

Our theoretical model, which includes a feedback equation for the control parameter of the system, describes well the dynamics observed experimentally. When poised in the chaotic regime, the system shows an enhanced sensitivity to weak stimulus. We therefore propose that hair cells of the auditory system harness the presence of chaos to achieve high sensitivity. We further hypothesize that chaotic dynamics may be a ubiquitous feature of nonlinear biological systems, which typically exhibit many degrees of freedom. It is therefore important to understand the impact of chaos on the sensory perception in living systems. Future work entails developing experimental techniques for modulating the degree of chaos in bundle dynamics, to assess the impact of chaoticity on the biological sensitivity of detection.

## Methods

### Experimental Techniques

Experiments were performed *in vitro* on hair cells of the amphibian sacculus, an organ specializing in low-frequency air- and ground-borne vibrations. Sacculi were excised from the inner ears of North American bullfrogs (*Rana catesbeiana*), and mounted in a two-compartment chamber^5^. Spontaneously oscillating hair bundles were accessed after digestion and removal of the overlying otolithic membrane^7^. All protocols for animal care and euthanasia were approved by the UCLA Chancellor’s Animal Research Committee in accordance with federal and state regulations. To deliver a stimulus to the hair bundles, we used glass capillaries that had been pulled with a micropipette puller. These elastic probes were treated with a charged polymer that improves adhesion to the hair bundle. Innate oscillations persisted after the attachment of probes with stiffness coefficients of ~100 *μ*N/m. The piezoelectric actuator was controlled with LabVIEW to apply sinusoidal stimulation of varying amplitudes for selected constant frequencies. Hair bundle motion was recorded with a high-speed camera at frame rates of 500 Hz or 1 kHz. The records were analysed in MATLAB, using a centre-of-pixel-intensity technique to determine the mean bundle position in each frame. Typical noise floors of this technique, combined with stochastic fluctuations of bundle position in the fluid, were 3–5 nm. Figure (1a) shows representative traces of active bundle motion, subject to mechanical forcing.

### Data availability

The data supporting the findings of this study are available within the article and its supplementary material file. Raw datasets generated during the current study are available from the corresponding author on reasonable request.

## Electronic supplementary material

Supplementary information
