# Supplementary material for: Chaotic Dynamics of Inner Ear Hair Cells
Source: Sci Rep. 2018 Feb 20;8:3366. doi: 10.1038/s41598-018-21538-z (PMC5820366; doi:10.1038/s41598-018-21538-z)
Supplement: Supplementary file 1 — Supplementary information [file 41598_2018_21538_MOESM1_ESM.pdf]

Supplemental Material for: Chaotic Dynamics of Inner Ear Hair Cells

Justin Faber[1] and Dolores Bozovic[1,2]
[1]*Department of Physics & Astronomy and* [2]*California NanoSystems Institute,*
University of California,
Los Angeles, California 90095, USA

Simulated Control Data Sets

We use several simulated data sets as controls (figure S1). Telegraphic noise was simulated by numerically solving the Langevin equation with a quartic potential,

$$\lambda \frac{dx}{dt} = \eta(t) - \frac{dU(x)}{dx}, \tag{1}$$

where

$$U(x) = ax^4 + bx^2. \tag{2}$$

We use $a = \frac{1}{4}$, $b = -5$, and $\lambda = 1$. For $\eta(t)$, we use Gaussian white noise with a standard deviation of 5.

As another control, we numerically solve the normal form equation for the Hopf bifurcation, up to cubic order,

$$\frac{dz}{dt} = (\mu - i\omega_0 + A|z|^2)z + \eta_z(t), \tag{3}$$

with $A = 10$, $\mu = \omega_0 = 1$, and Gaussian white noise added to real and imaginary parts of z ($\eta_z = \eta_x + i\eta_y$), each with standard deviation of 0.1.

As a third control, we use a sine wave and superpose Gaussian white noise (signal-to-noise ratio = 5).

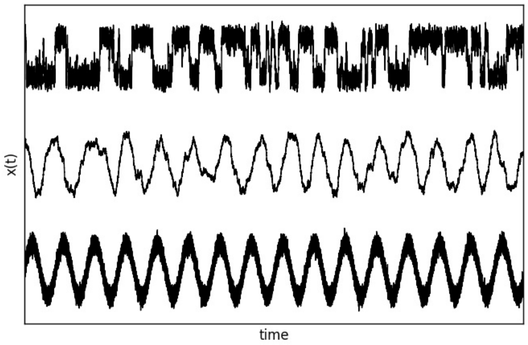

FIG. S1. Data sets used as controls. Telegraphic noise (top), suprcritical Hopf system (middle), and sine wave with added noise (bottom).

Test for Determinism

To confirm that a deterministic component is present in the spontansouly oscilating hair bundle, we performed the direct test for determinism [1]. This method relies on reconstructing the phase space using delayed coordinates and comparing the direction of local flow throughout the phase space. We divide the phase space into hypercubes and construct unit vectors from the point at which a trajectory enters a given hypercube to that at which it exits. For a purely stochastic system, the sum of unit vectors associated with a given hypercube decreases as more vectors are added. However, for deterministic systems, these unit vectors generally point in the same direction. As a result, the sum should remain close to unity as vectors are added.

We used 5 embedding dimensions and divided the phase space into 10 bins per dimension. Similar results were obtained for choices of 4 and 6 embedding dimensions and choices of 4, 7, and 12 bins. Using Welch's unequal variances t-test, The raw data is distinguishable from the phase-shuffled surrogate data with P values ranging from $0.008 - 0.015$ for number of passes $2 - 50$. Welch's t-test is appropriate since the two distributions are of different sample size and variance. Further, we demonstrate that this method is robust when a deterministic signal is contaminated with noise (figure S2).

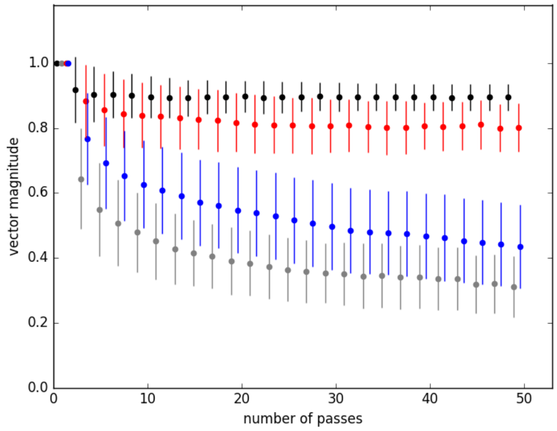

FIG. S2. Direct test for determinism on a spontaneously oscillating hair bundle. Red and blue points represent the raw data and phase-shuffled surrogate data, respectively ($N = 1.8 \times 10^5$ data points). Black points represent a sine wave with additive white noise (signal-to-noise ratio = 5). Grey points represent telegraphic noise. Points and error bars represent the mean and standard deviation across all occupied hypercubes in the phase space.

False Nearest Neighbour Test

We perform the false nearest neighbour test as another way of estimating the embedding dimension of the hair cell system. This method relies on reconstructing the phase space using delayed coordinates. When the embedding dimension is too low, not all points that lie near each other will do so because of the dynamics. Instead, some may be neighbours because the structure of the attractor has been projected into a lower dimensional space. When the embedding dimension is high enough to fully unfold the attractor, there will be no more false nearest neighbours. However, if the signal is contaminated by stochastic processes, there will always remain some fraction of false nearest neighbours. With experimental data contaminated with noise, we look for a plateau in the fraction of false nearest neighbours as the embedding dimension is increased. Using the method described in [2, 3] on a long recording of spontaneous hair bundle oscillations, we find that the attractor is fully unfolded with six embedding dimensions (figure S3), a result consistent with our previous finding. Use used $R_{tol} = 15$ and $A_{tol} = 2.5$ as the thresholds for identifying false nearest neighbours (see [2]). However, the optimal embedding did not change upon varying either of these parameters ($R_{tol} = 10$ or 20 and $A_{tol} = 2$ or 3). The embedding dimension was negligibly affected by weak or moderate stimulus. Only during strong stimulus, ∼15 pN, was the embedding dimension reduced.

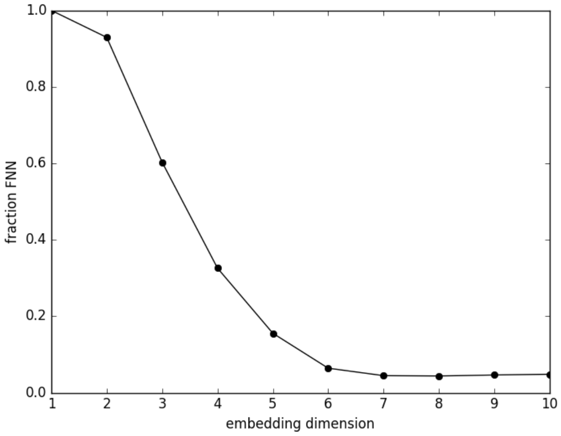

FIG. S3. False nearest neighbour test performed on a spontaneously oscillating hair bundle. The same full length data set ($N = 1.8 \times 10^5$ data points) was used in figures 2 and S3.

Stimulus Near the Resonance Frequency

When hair bundles are stimulated near resonance, the Poincaré maps do not exhibit quasiperiodic transitions. Instead, as the forcing amplitude increases, the cloud in the I_n–I_{n+1} plane shrinks in size and collapses onto a point (figure S4). K-entropy decreases with increasing amplitude of the drive, while information transmission increases (figure S5), comparable to the dependence observed with below-resonance stimulus. As expected, the threshold for detecting weak stimulus is lower for the on-resonance case.

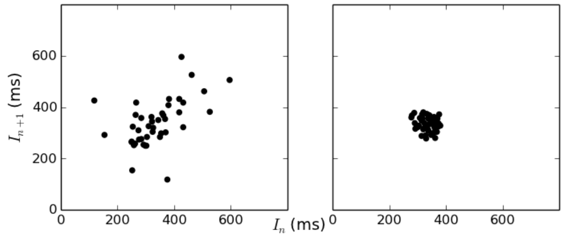

FIG. S4. Poincaré maps representing spontaneous oscillations of hair bundles (left) and hair bundle response to a sinusoidal stimulus of ∼5 pN applied near the resonance frequency (right).

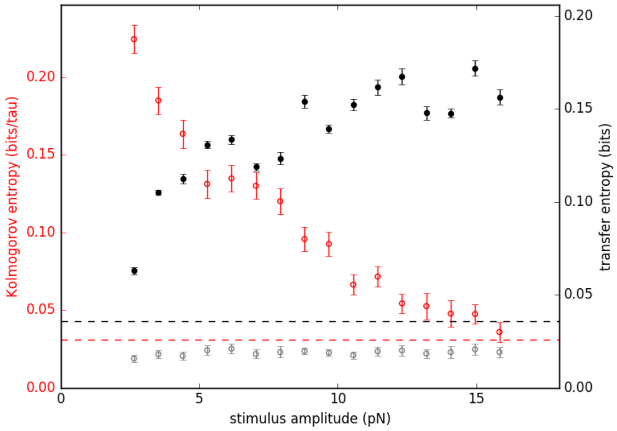

FIG. S5. The Kolmogorov entropy of an oscillatory hair bundle subject to a sinusoidal drive (red), transfer entropy from the stimulus to the bundle (black-filled), and transfer entropy from bundel to stimulus (black-open) plotted as a function of the stimulus amplitude. Noise floors are indicated by horizontal dashed lines. Stimulus frequency was selected to be near the resonance frequency of the hair bundle.

Stimulus Above the Resonance Frequency

A different type of transition from chaos to order is observed when the stimulus is applied at frequencies above resonance. Rather than displaying a ring, the points on the Poincaré maps cluster into regions corresponding to integer multiples of the resonance frequency, indicative of high-order mode-locking (figure S6). Rather than migrating around the edge (as in the case of a quasiperiodic transition), consecutive points cross over the centre. Upon higher amplitudes of the drive, the cluster corresponding to 1:1 mode-locking dominates, and other clusters vanish. K-entropy decreases with increasing amplitude of the drive, while information transmission increases (figure S7), comparable to the dependence observed with below-resonance stimulus. However, K-entropy initially increases for weak forcing. We attribute this initial increase in K-entropy to flicker between 1:1 and 2:1 modes in the phase-locking of the bundle to the stimulus. We note that this flicker corresponds to phase-slips, which have been observed and characterised in prior studies [4]. We have shown that the hair bundle exhibits low-dimensional chaos in the absence of stimulus (see main text figures 2 and 4), however we are unable to identify the route from chaos to order for above-resonance stimulus using Poincaré maps.

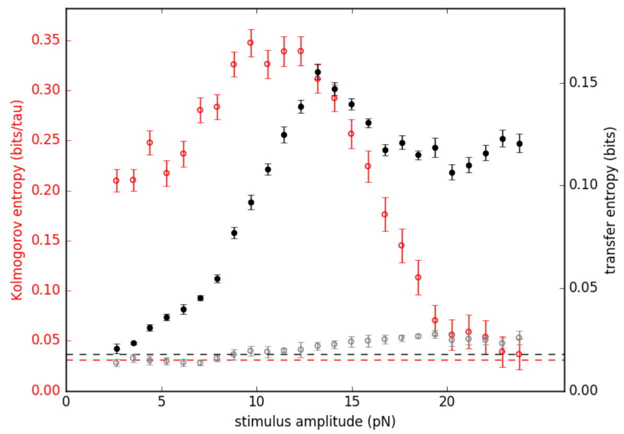

FIG. S7. The Kolmogorov entropy of an oscillatory hair bundle subject to a sinusoidal drive (red), transfer entropy from the stimulus to the bundle (black-filled), and transfer entropy from bundel to stimulus (black-open) plotted as a function of the stimulus amplitude. Noise floors are indicated by horizontal dashed lines. Stimulus frequency was above the resonance frequency of the hair bundle ($\omega \sim \frac{5}{3}\omega_0$).

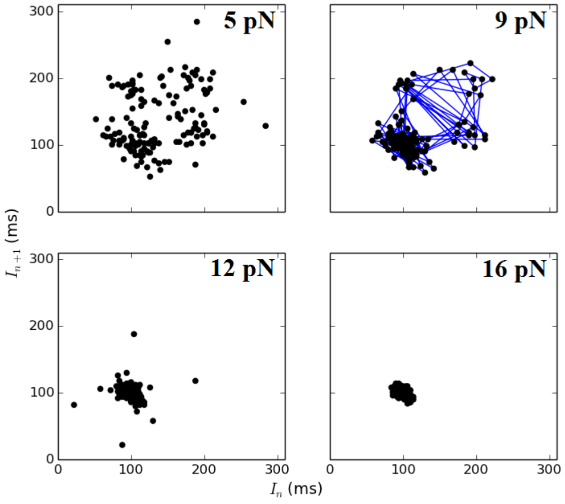

FIG. S6. Poincaré maps representing oscillations of hair bundles driven by a sinusoidal stimulus above the resonance frequency ($\omega \sim \frac{5}{3}\omega_0$). The four clusters of points indicate a flicker between 1:1 and 1:2 mode-locking. Blue lines connect consecutive points in the series.

Theoretical Model

A sinusoidal stimulus of linearly increasing amplitude was applied to the theoretical model. With $\alpha > 0$, the model exhibits a quasiperiodic transition from chaos to order as forcing amplitude is increased (figure S8).

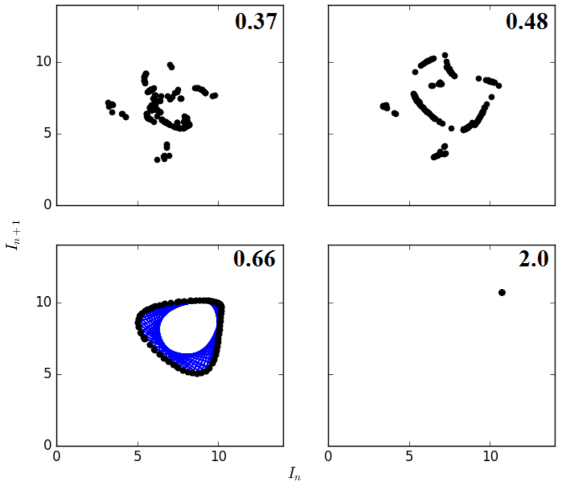

FIG. S8. (Model) Poincaré maps representing oscillations of hair bundles driven by a sinusoidal stimulus below the resonance frequency ($\omega \sim \frac{2}{3}\omega_0$), for a range of forcing amplitudes, as indicated in the top right corners. Blue lines connect consecutive points in the series. $x_0 = 0.4$, $k_{on} = \frac{5}{12}$, $k_{off} = \frac{7\pi}{12\arccos(0.4)}$, $A = 10$, and $\alpha = 0.15$.

Poincaré Maps and Stochastic Processes

Our analysis of experimental data indicate that the quasi-periodic regime observed at moderate stimulus levels corresponds to the torus-breakdown route to chaos. This finding implies that the cloud structure observed at weak stimuli contains an underlying chaotic attractor. Here, we preform numerical simulations on three systems, to obtain corresponding Poincaré and angle maps, and verify that different results are obtained for non-chaotic systems with superposed noise.

First, we obtain time traces of telegraphic noise, generated by simulating a particle in a quartic potential driven by stochastic noise and a superposed sinusoidal force. For weak stimuli, the interval Poincaré maps of telegraphic noise form a cloud, and at strong stimuli, the maps collapse onto a point. In the intermediate range stimulus amplitudes, the system does not exhibit a ring-like structure, but rather shows complex mode-locking behavior (figure S9). 1:1, 1:2, and 1:3 mode-locking can be seen in the Poincaré map. Next, we tested whether simulations of the supercritical Hopf oscillator with superposed noise show similar behavior to the experimental records. While the Poincaré maps do exhibit similar quasiperiodic behavior (figure S10), the corresponding circle map forms an invertible function indicative of a non-chaotic system. We obtained Poincaré and circle maps similar to figure S10 for the supercritical Hopf oscillator with noise superposed on the phase variable, or parametric noise superposed on the control parameter μ.

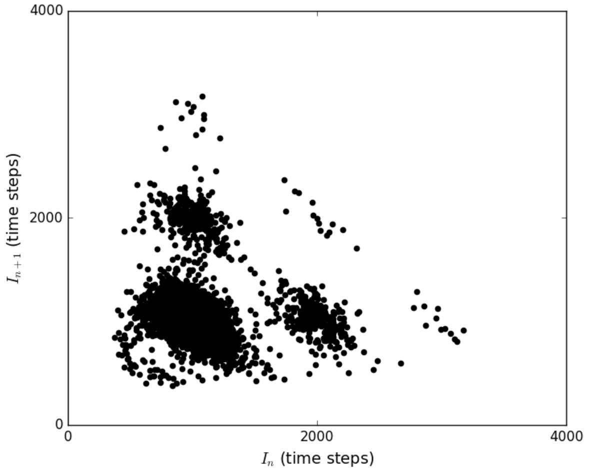

FIG. S9. Poincaré map representing telegraphic noise generated by simulating a particle in a quartic potential driven by white noise and sinusoidal forcing.

We observed the same regime of quasiperiodicity with a corresponding invertible circle map, for a driven, noisy harmonic oscillator. These simulations demonstrate that this technique is robust in the presence of stochastic processes, as an invertible circle map is not consistent with torus breakdown. Hence, it confirms that the structure in the Poincaré maps of our experimental recordings are a manifestation of a chaotic attractor, rather than a two-dimensional system in the presence of noise.

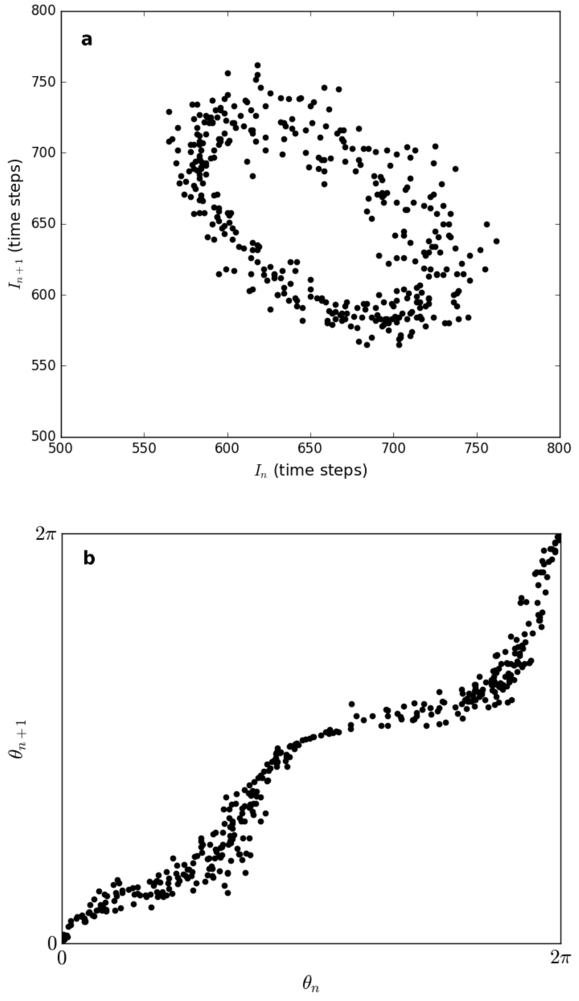

FIG. S10. (**a**) Poincaré map representing a simulation of the supercritical Hopf system driven by white noise and sinusoidal forcing below the resonance frequency ($\omega \sim \frac{2}{3}\omega_0$). (**b**) Corresponding Circle Map.

[1] D. Kaplan and L. Glass, Phys Rev Lett **68**, 427 (1992).
[2] M. Kennel, R. Brown, and H. Abarbanel, Physical Review A **45**, 3403 (1992).
[3] M. B. Kennel and H. D. I. Abarbanel, Physical Review E - Statistical, Nonlinear, and Soft Matter Physics **66**, 1 (2002).

[4] Y. Roongthumskul, R. Shlomovitz, R. Bruinsma, and D. Bozovic, Physical Review Letters **110**, 1 (2013).
